# Event-Level Identification of Sleep Apnea Using FMCW Radar

**DOI:** 10.3390/bioengineering12040399

**Published:** 2025-04-08

**Authors:** Hao Zhang, Shining Bo, Xuan Zhang, Peng Wang, Lidong Du, Zhenfeng Li, Pang Wu, Xianxiang Chen, Libin Jiang, Zhen Fang

**Affiliations:** 1 Aerospace Information Research Institute, Chinese Academy of Sciences (AIRCAS), Beijing 100094, China; zhanghao190@mails.ucas.ac.cn (H.Z.); wangpeng01@aircas.ac.cn (P.W.); lddu@mail.ie.ac.cn (L.D.); lizhenfeng@aircas.ac.cn (Z.L.); wupang@aircas.ac.cn (P.W.); chenxx@aircas.ac.cn (X.C.); 2 School of Electronic, Electrical and Communication Engineering, University of Chinese Academy of Sciences, Beijing 100049, China; 3 Department of Intensive Care Unit, Peking University Third Hospital, Beijing 100096, China; boshining@bjmu.edu.cn; 4 Beijing Tian Tan Hospital, Capital Medical University, Beijing 100162, China; ming_nz@163.com; 5 Beijing Tongren Eye Center, Beijing Tongren Hospital, Capital Medical University, Beijing 100730, China; 6 Personalized Management of Chronic Respiratory Disease, Chinese Academy of Medical Sciences, Beijing 100190, China

**Keywords:** sleep apnea, FMCW radar, event-level detection

## Abstract

Sleep apnea, characterized by its high prevalence and serious health consequences, faces a critical bottleneck in diagnosis. Polysomnography (PSG), the gold standard, is costly and cumbersome, while wearable devices struggle with quality control and patient compliance, rendering them as unsuitable for both large-scale screening and continuous monitoring. To address these challenges, this research introduces a contactless, low-cost, and accurate event-level sleep apnea detection method leveraging frequency-modulated continuous-wave (FMCW) radar technology. The core of our approach is a novel deep-learning model, built upon the U-Net architecture and augmented with self-attention mechanisms and squeeze-and-excitation (SE) modules, meticulously designed for the precise event-level segmentation of sleep apnea from FMCW radar signals. Crucially, we integrate blood oxygen saturation (SpO_2_) prediction as an auxiliary task within a multitask-learning framework to enhance the model’s feature extraction capabilities and clinical utility by capturing physiological correlations between apnea events and oxygen levels. Rigorous evaluation in a clinical dataset, comprising data from 35 participants, with synchronized PSG and radar data demonstrated a performance exceeding that of the baseline methods (Base U-Net and CNN–MHA), achieving a high level of accuracy in event-level segmentation (with an F1-score of 0.8019) and OSA severity grading (91.43%). These findings underscore the significant potential of our radar-based event-level detection system as a non-contact, low-cost, and accurate solution for OSA assessment. This technology offers a promising avenue for transforming sleep apnea diagnosis, making large-scale screening and continuous home monitoring a practical reality and ultimately leading to improved patient outcomes and public health impacts.

## 1. Introduction

Obstructive sleep apnea (OSA) is a common sleep disorder characterized by recurrent episodes of apnea or hypopnea during sleep. OSA is not merely associated with daytime sleepiness, fatigue, and impaired concentration. Crucially, it is recognized as an independent risk factor for several severe conditions, including cardiovascular diseases (e.g., hypertension, coronary artery disease, and heart failure), metabolic disorders (e.g., diabetes and obesity), and neurocognitive impairments (e.g., cognitive dysfunction and memory decline). OSA also significantly elevates the risks of traffic accidents and all-cause mortality [1]. With rates of aging and and obesity continuously increasing in the global population, the prevalence of OSA is rising annually, evolving into a growing public health challenge. It is estimated that approximately 936 million adults globally suffer from OSA, with 425 million cases classified as moderate–severe [1]. This vast affected population underscores the extensive impact of OSA and its significant threat to human health. Therefore, early and accurate diagnosis, coupled with timely and effective intervention, is essential for enhancing patient prognosis and alleviating the social and clinical burden associated with OSA.

The primary pathophysiology of OSA involves repeated upper airway obstruction during sleep, with its development and progression influenced by a complex interplay of factors, including anatomical structure, neural regulation, and muscle function [1]. According to the Apnea–Hypopnea Index (AHI), OSA is typically categorized into mild (AHI, 5–15 events/hour), moderate (AHI, 15–30 events/hour), and severe (AHI, >30 events/hour) levels [2]. Polysomnography (PSG) serves as the acknowledged gold standard for OSA diagnosis. This comprehensive diagnostic modality entails the synchronized measurement of an array of physiological signals within a sleep laboratory environment, encompassing electroencephalography (EEG), electrocardiography (ECG), nasal and oral airflows, respiratory effort (both thoracic and abdominal), and blood oxygen saturation (SpO_2_). Diagnostic conclusions are subsequently reached through the manual scoring of PSG recordings by trained professionals, adhering to the American Academy of Sleep Medicine (AASM) scoring manual [2]. However, PSG has inherent limitations that are becoming increasingly apparent. PSG examinations are costly, are complex to operate, require specialized medical personnel in sleep laboratories, and are uncomfortable for patients, who often exhibit a significant first-night effect [3]. These inherent limitations make PSG unsuitable for large-scale OSA screening and long-term monitoring needs.

Despite the high number of OSA patients, current screening and diagnostic methods present a prominent contradiction. First, a mismatch exists between the vast patient population and limited, inconvenient screening methods. Traditional PSG equipment is expensive, complex to operate, and highly dependent on specialized personnel, severely limiting its accessibility and resulting in a diagnosis rate far lower than the actual prevalence [4]. Second, a conflict arises between the need for the long-term follow-up of the OSA treatment efficacy and the lack of economical, comfortable, and convenient monitoring tools. Although existing portable monitoring devices have improved the convenience of home monitoring to some extent, they still suffer from issues such as poor patient compliance and difficulties in ensuring sustained comfort and convenience. Consequently, they cannot fully meet the demand for long-term, convenient, and accurate efficacy evaluation in home settings [5]. Therefore, developing cost-effective, convenient, accurate, and comfortable OSA monitoring technologies to improve early diagnosis rates and meet the needs for long-term monitoring and treatment efficacy evaluation has become a critical technical challenge in the field of OSA management and urgently needs to be addressed. To clearly illustrate the tradeoffs between different technical solutions for OSA monitoring, we summarize the performance comparison of existing mainstream technologies in Table 1.

In an effort to address some of the aforementioned limitations and enhance the accessibility and convenience of OSA diagnosis, portable PSG devices have emerged in recent years. These devices typically collect only a limited number of physiological signals, such as ECG, photoplethysmography (PPG), and respiratory signals, and employ automatic analysis algorithms for OSA diagnosis, lessening the dependence on specialized equipment and personnel to some degree [4]. Compared to traditional PSG, portable PSG devices significantly improve convenience and reduce costs, making home-based OSA monitoring feasible [6]. For example, portable-ECG-based devices, leveraging deep-learning algorithms, have achieved high accuracy in OSA detection [7]. Portable PSG, despite its advancements, is fundamentally limited by its “wearable” nature, and, thus, inherent drawbacks persist. Patients are still required to wear various sensors; thus, comfort issues remain, and long-term wear compliance is difficult to guarantee. Simultaneously, the signal quality is susceptible to factors such as wearing position, tightness, and patient condition, making it challenging to ensure signal stability and reliability [3]. Therefore, although portable PSG devices have made progress in improving the accessibility of OSA diagnosis, they remain essentially “contact-based” monitoring solutions. The discomfort inherent to wearable sensors and the persistent challenge of ensuring reliable signal quality continue to impede their broader clinical application.

**Table 1:** Comparison of various sleep apnea detection studies.

Type	Study	Signal	Model	Comfort	Quality Control	Cost	Accuracy	F1
Contact	[7]	ECG	CNN	Medium	Medium	Low	90.64%	87.57%
	[8]	PPG	SVM	Medium	Hard	Low	75.10%	76.03%
	[9]	Resp	CNN	Medium	Hard	Medium	95.67%	66.55%
	[10]	Flow	CNN	Poor	Hard	Medium	96.55%	83.91%
Contactless	[11]	Piez	CNN	Best	Medium	Low	84.68%	52.48%
	[12]	Piez	CNN	Best	Medium	Low	97.50%	74.70%
	[5]	FMCW	RF	Best	Easy	Low	95.53%	70.21%
	[13]	FMCW	CRNN	Best	Easy	Low	78.40%	68.80%
	[14]	UWB	CNN	Best	Easy	High	88.89%	90.95%

The rise in contactless monitoring technology offers a highly promising solution to effectively address the limitations of portable PSG devices. This technology can acquire physiological signals without direct contact with the human body, fundamentally eliminating the discomfort associated with “wearing”. It offers the potential to genuinely address the comfort and compliance issues of long-term monitoring [11]. For instance, monitoring solutions based on piezoelectric ceramic mattresses and radar have attracted significant attention, while piezoelectric ceramic solutions are relatively low in cost; they are susceptible to environmental interference, exhibit poor stability, and are less effective at capturing nuanced physiological information [12]. Furthermore, piezoelectric ceramic sensors are known to exhibit aging and performance degradation over extended use [5]. Magnetic technology offers another promising non-contact approach with unique dual-function capabilities. Advanced magneto-impedance (MI) sensors can detect the brain’s magnetic fields with pico-Tesla resolution at room temperature [15], while magnetic stimulation systems can simultaneously provide arousal retention effects without the sleep rebound phenomenon observed in other stimulation methods [16]. This complementary capability of using magnetic fields for both monitoring and intervention represents an integrated approach to sleep monitoring and management. In contrast, radar-based monitoring technologies demonstrate greater advantages. These include ultra-wideband (UWB) radar and frequency-modulated continuous-wave (FMCW) radar. UWB radar [14], with its excellent signal quality and efficient signal-processing algorithms, has achieved a high accuracy rate (88.89%) and F1-score (90.95%) in OSA detection. In addition to targeting apnea itself, there are studies using UWB radar for the identification of sleep postures, which is necessary for apnea diagnosis because the occurrence of obstructive apnea is strongly correlated with posture [17]. However, the supply of integrated UWB radar chips is limited, and costs are high. FMCW radar, on the other hand, because of its advantages of low cost, low power consumption, ease of integration, a robust industrial ecosystem, and wide chip availability, shows great application potential in consumer electronics and health-monitoring fields. However, we note that existing FMCW-radar-based OSA studies mostly employ fixed-length segments or sliding windows for analysis, providing only segment-level or short-duration OSA risk prediction and failing to achieve the precise segmentation of apnea events and event-level diagnostic information [5,13]. Clinical diagnosis and treatment decisions typically require detailed insights into aspects like the type, duration, and severity of apnea events. The lack of event-level diagnostic capability significantly limits the clinical application value of existing radar technologies in OSA. Moreover, current FMCW-radar-based OSA detection methods still offer opportunities for enhanced performance, particularly in sensitivity and F1-score.

This study aims to address a central question: how event-level sleep apnea diagnosis can be achieved using contactless technology while maintaining diagnostic quality comparable to that of clinical standards. Specifically, we transform the segment-based classification task into a time-series segmentation task to calculate the probability of an abnormal respiratory event at each time point to determine the exact start and end times of the event. In addition, blood oxygen saturation prediction is introduced to the multitask-learning framework as an auxiliary task to provide the model with more fine-grained supervision signals and improve the detection ability of abnormal respiratory events. Validated with real clinical data, this study fills the gap in existing FMCW-radar-based monitoring methods by providing clinically necessary event-level diagnostic information.

## 2. Methods

### 2.1. System Overview

In this chapter, we present a step-by-step account of the implementation of an FMCW-radar-based system for sleep apnea event segmentation at the event level.

**Figure 1:**
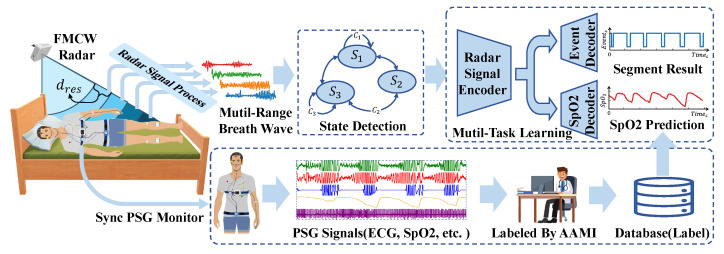
Overview of the FMCW-radar-based sleep apnea monitoring system.

The comprehensive system architecture is depicted in Figure 1. As illustrated, the system is designed for monitoring subjects’ sleep throughout the night. The FMCW radar is positioned at the bedside and tilted downward to ensure the subject’s upper body is within the radar beam’s coverage area. Subsequently, basic radar signal processing is applied to the echoes reflected from the subject to derive multichannel respiratory signals. A human state classifier, based on a state machine paradigm, is employed for signal segregation. Our respiratory event segmentation model exclusively processes segments classified as “lie in bed”. In addition to the event segmentation output, the model is designed with a blood oxygen saturation prediction branch, which helps the radar signal encoder to better encode the features related to respiration and provides synchronous blood oxygen saturation prediction results. Concurrently, subjects underwent standard PSG for reference. Following the monitoring, professional sleep physicians manually annotated the PSG data according to the AASM criteria to derive sleep event labels. The resulting labels, alongside the raw PSG data, were then compiled into a database for subsequent training and evaluation purposes.

### 2.2. Radar Principles

**Figure 2:**
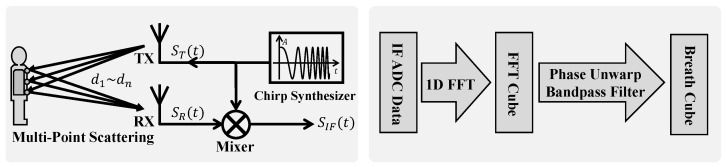
Schematic diagram of the FMCW radar structure and basic signal-processing flow.

In previous studies on heart rate [18] and respiration monitoring [5,13,19], the single-point scattering model, treating the human body as a point target, has been widely adopted. However, our experimental observations reveal that this simplification is insufficient to fully characterize the complex nature of radar echo signals from the human body. Specifically, we have observed significant amplitudinal variations in radar echoes, alongside the expected phase modulation because of respiratory motion, and the signal energy is distributed across multiple range bins. Although the single-point model can account for the phase modulation, it fails to explain the amplitudinal modulation phenomenon and exhibits discrepancies in phase characteristics compared to those of real signals. To address these limitations and more accurately model radar reflections from respiratory motion, this study introduces the multipoint scattering model as shown in Figure 2.

In contrast to the single-point model, the multipoint scattering model represents the human body as a collection of *N* independent scattering points, ${\left\{P_{n}\right\}}_{n=1}^{N}$, distributed across areas such as the chest and abdomen. The distance, $d_{n}\left(t\right)$, between each scattering point, $P_{n}$, and the radar is modulated by respiratory motion, expressed as $d_{n}\left(t\right)=d_{n,0}+b_{n}\left(t\right)$, where $d_{n,0}$ is the static distance, and $b_{n}\left(t\right)$ is the respiratory-motion-induced distance variation. It is important to note that the respiratory motion, $b_{n}\left(t\right)$, can vary among different scattering points, especially under pathological breathing conditions, like paradoxical breathing in OSA. The FMCW radar transmits a chirp signal with linearly increasing frequency as follows:

(1) $$S_{T}\left(t\right)=A_{T}e^{\pi St^{2}+2\pi f_{0}t},\;0\leq t\leq T_{c}$$

where $A_{T}$, *S*, and $f_{0}$ are the transmitted signal’s amplitude, chirp rate, and initial frequency, respectively. The chirp rate is defined as $S=\frac{B}{T_{c}}$. The received signal is the superposition of the reflections from all the scattering points. After mixing and sampling, the FMCW radar yields an intermediate-frequency (IF) signal as follows:

(2) $$\begin{array}{c} S_{IF}\left(t\right)=\sum_{n=1}^{N}\alpha_{n}A_{T}e^{2\pi S\frac{2d_{n}\left(t\right)}{c}t+2\pi f_{0}\frac{2d_{n}\left(t\right)}{c}},\;\tau_{max}\leq t\leq T_{c} \end{array}$$

where $\alpha_{n}$ and $d_{n}$ are the reflection coefficient and distance to the *n*th scattering point. Equation (2) reveals that respiratory motion modulates the $S_{IF}$ signal in several key aspects:

- Range information: The frequency component, $2\pi S\frac{2d_{n}\left(t\right)}{c}t$, is directly proportional to the distance, $d_{n}\left(t\right)$, from the scattering point. Therefore, the frequency of the IF signal encodes the range information of the scatterers. By performing frequency analysis (FFT) on the IF signal, we can resolve reflections from different ranges, forming the basis for the range profile and, subsequently, the range–time matrix;
- Phase sensitivity to micro-motion: The phase term, $2\pi f_{0}\frac{2d_{n}\left(t\right)}{c}=\frac{4\pi }{\lambda }d_{n}\left(t\right)$, is directly modulated by the distance, $d_{n}\left(t\right)$, and thus by the respiratory-motion-induced distance variation, $b_{n}\left(t\right)$. Given the short wavelength, $\lambda =c/f_{0}$, of millimeter-wave radar (e.g., λ=5mm for 60 GHz radar), even subtle respiratory movements induce significant phase changes. This high sensitivity makes the phase an extremely effective indicator of respiratory motion;
- Amplitudinal modulation because of multipoint interference: The total IF signal, $S_{IF}$, is the vector sum of reflected signals from *N* scattering points. Because of variations in distances, $d_{n}$, and respiratory motions, $b_{n}\left(t\right)$, among different points, the phases of individual reflected signals differ. As respiration occurs, changes in $d_{n}\left(t\right)$ lead to temporal variations in the phases. The amplitude of the vector sum is influenced by these phasic relationships. Constructive interference, resulting from phase variations, increases the overall signal amplitude, while destructive interference decreases it. Consequently, in the multipoint scattering model, the IF signal amplitude is modulated by respiratory motion. This amplitudinal modulation, arising from the coherent superposition of multipoint reflections, contains richer respiratory information, including motion disparities, across different body regions.

Single chirp signals are extremely short in duration (microseconds), allowing us to consider scatterers as stationary within a chirp. However, to continuously monitor respiratory-motion-induced displacement, we need to transmit chirp signals repeatedly. By arranging the range of samples from consecutive chirps in chronological order, we construct the range–time matrix. Each column of this matrix represents the range profile within a single chirp, and each row tracks the signal variation from a specific range bin over time. The range–time matrix thus captures the temporal evolution of radar reflections from different distances, providing the foundation for respiratory signal extraction and analysis. According to the preceding analysis, the modulation effects of respiration on the radar signal will be manifested in the phasic and amplitudinal information within the range–time matrix.

**Figure 3:**
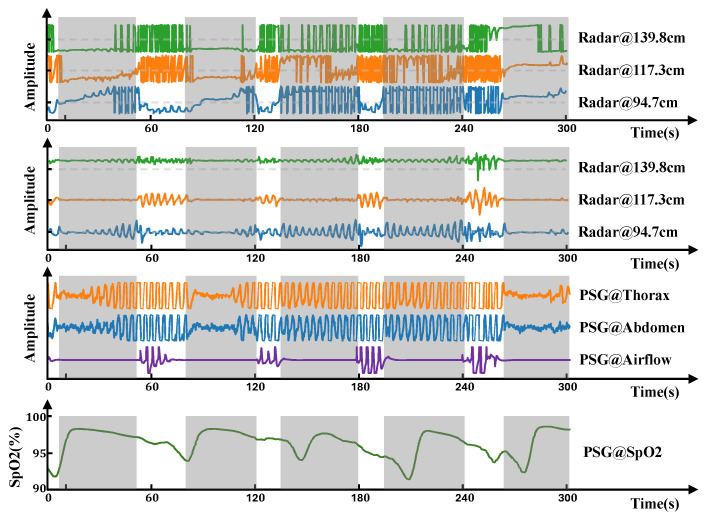
A typical radar respiratory signal from different range bins with a length of 5 min, indicating that the person is experiencing repeated apnea events. From top to bottom are the original IF phase signal, the processed IF phase signal, the synchronized chest and abdominal respiratory banding acquired from PSG, the nasal airflow signal, and the blood oxygen saturation signal.

The phasic information in the range–time matrix carries valuable respiratory signals. However, the directly acquired phase appears wrapped, meaning the true phasic variations are folded into the [−π,π] interval because of the modulo-2π operation, resulting in phase discontinuities or jumps, as depicted in Figure 3. These phase jumps obscure the actual amplitude of the respiratory motion. To recover the original continuous phasic information, phase unwrapping is necessary. This process reconstructs a continuous phase signal by adding or subtracting multiples of 2π at phase jump points. Even after phase unwrapping, the signal may still exhibit baseline drift, often caused by accumulated phase errors or low-frequency noise. To eliminate this baseline wander, isolate the respiratory component, and filter out low-frequency interference, we employ high-pass filtering. In this study, we used a third-order Butterworth high-pass filter with a cutoff frequency of 0.1 Hz. The resulting respiration signal, after filtering, is shown in Figure 3, with gray shaded areas indicating apnea events. From Figure 3, we can see that reflected signals from different range bins contain distinct respiratory information. The respiratory signal obtained by radar shows synchronous changes with the respiratory band and airflow and is closer to the respiratory airflow signal in morphology. This spatial diversity in respiratory signals across range bins provides rich and informative input for subsequent respiratory event segmentation models and detailed respiratory analysis.

### 2.3. People’s Status Detection

This section details our designed bed occupancy detection method, which aims to identify human states in real time with low computational cost on the device side and, ultimately, accurately recognize the “Lie-in-Bed” state to trigger subsequent high-computational sleep event segmentation algorithms. Our approach is based on the energy analysis of radar signals from different distance zones combined with a state machine to determine the bed occupancy status.

**Figure 4:**
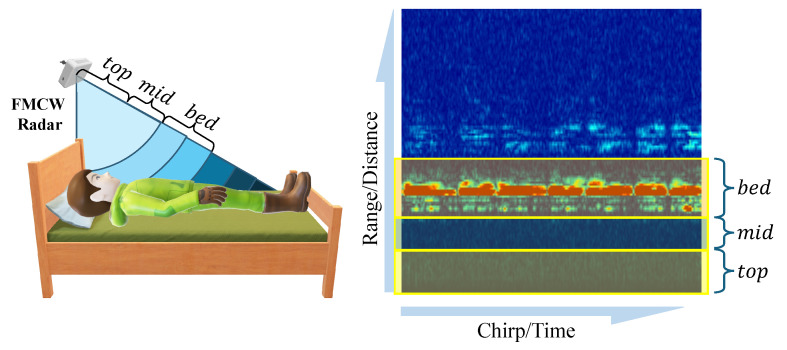
Range segmentation for bed occupancy detection.

Our method initially divides the radar detection range into three key zones: top, mid, and bed layers. These are illustrated in Figure 4. The bed layer primarily corresponds to the distance range of the bed surface, used for monitoring human reflection signals on the bed. The top layer corresponds to the space above the human body, assisting in determining whether a person is standing or sitting beside the bed. The mid layer serves as a buffer, designed to increase the distance between the bed and top layers, thereby reducing the number of false positives.

Specifically, our system first undergoes calibration after installation, where a subject lies on the bed, and we select the distance unit with the strongest reflection energy as the central reference point for the bed layer. According to the distance resolution of the radar used (4.51 cm), we define the bed layer as nine distance units (approximately 40 cm thickness), sufficient to cover the human body’s thickness; the mid layer is defined as five distance units (approximately 22.55 cm); and the top layer contains the remaining distance units. The radar radiates toward the bed surface at an inclined angle, a design that allows the bed layer to cover the entire bed’s surface area, ensuring that reflection signals remain within the corresponding zones even when the body’s posture changes (such as while turning over). This calibration-based partitioning method also gives the system good adaptability to variations in bed height.

**Figure 5:**
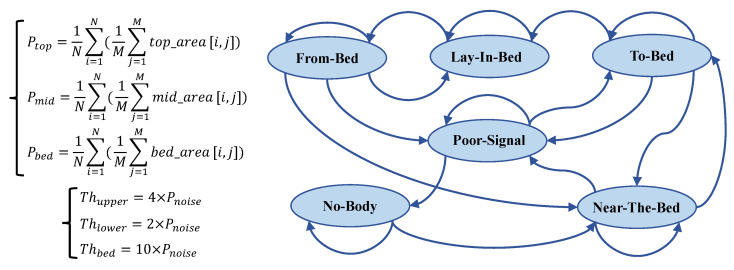
State machine for bed occupancy detection with range-based power-thresholding logic.

Subsequently, we calculate the power level for each distance zone, denoted as $P_{top}$, $P_{mid}$, and $P_{bed}$, respectively. These power levels are obtained by averaging the IF signal’s amplitude within the corresponding areas (*top_area*, *mid_area*, and *bed_area*) in the range–time heat map, as shown in Figure 5. $P_{top}$, $P_{mid}$, and $P_{bed}$ represent the energy intensity of the radar signal in different distance zones, effectively reflecting the presence of a human body at different spatial locations.

To achieve robust and accurate bed occupancy state detection, we design a state machine with six states, as depicted in Figure 5. The core of the state machine lies in its state definitions and transition rules. State transitions are driven by comparing the power levels of the three distance zones, $P_{top}$, $P_{mid}$, and $P_{bed}$, with predefined thresholds ($Th_{upper}$, $Th_{lower}$, and $Th_{bed}$). The state machine design incorporates the hysteresis comparison concept of a Schmitt trigger and introduces a debouncing mechanism to enhance robustness. The following list provides concise descriptions of each state and their primary transition logics:

- No Body: The baseline state, indicating no human presence detected. This state is maintained if $P_{bed}<Th_{upper}$. Transition to the Near-the-Bed state occurs if $P_{bed}\geq Th_{upper}$;
- Near the Bed: The initial detection of human proximity. State maintenance primarily depends on $P_{bed}$ and $P_{top}$. The key transitions include the following:
  – Transition to the BadSignal state occurs if $P_{bed}<Th_{bed}\wedge P_{top}<Th_{lower}\wedge P_{mid}<Th_{lower}$;
  – Transition to the To-Bed state occurs state if $P_{bed}\geq Th_{bed}\wedge P_{top}<Th_{upper}$;
- To Bed: Indicates that a person is possibly entering their bed and preparing to lie down. This state serves as a transition state, incorporating a 15-consecutive-frame (3 s) debouncing mechanism. Transition to the Lie-in-Bed state occurs if for 15 consecutive frames, $P_{top}<Th_{upper}\wedge P_{bed}\geq Th_{bed}$. Otherwise, a return to the Near-the-Bed or BadSignal state occurs;
- Lie in Bed: The core state, indicating the stable detection of a person lying in bed, triggering the sleep event segmentation algorithm. This state is maintained if $P_{top}<Th_{lower}\wedge P_{mid}<Th_{lower}\wedge P_{bed}\geq Th_{bed}$. Transition to the From-Bed state occurs if $P_{top}\geq Th_{lower}\vee P_{mid}\geq Th_{lower}\vee P_{bed}<Th_{bed}$;
- From Bed: Indicates that a person is possibly leaving their bed. A 15-consecutive-frame debouncing mechanism is also incorporated. The state maintenance and transition logic are more complex, primarily relying on changes in power levels across zones to determine whether to return to the Lie-in-Bed state, transition to the BadSignal state, or transition to the Near-the-Bed state;
- BadSignal: Indicates poor signal quality. This state may be entered when power levels across zones are generally low or because of state misjudgment. State transitions mainly depend on changes in $P_{bed}$ to determine whether to return to the No-Body state, maintain the BadSignal state, or transition to the To-Bed state.

This section has detailed the designed distance-based state machine for bed occupancy detection. This method effectively leverages the range resolution capability of FMCW radar, achieving robust, accurate, and detailed bed occupancy status detection through the analysis of radar signals’ energies in different distance zones, combined with reasonable threshold settings and state transition logic. The performance of this algorithm has been validated in clinical environments, demonstrating its reliability in real-world applications. Notably, the subsequent sleep event segmentation algorithm is triggered only when the state machine enters the Lie-in-Bed state, thereby effectively reducing the overall computational cost of the system while ensuring detection accuracy.

### 2.4. Model Design

In prior work, Li et al. [20] employed UWB signals as input to develop a CNN encoder coupled with a multihead self-attention mechanism for respiratory event prediction. Their model achieved impressive performance, with both the accuracy and F1-score exceeding 0.9 for second-by-second predictions. However, the downsampling inherent in their CNN encoder design presented a limitation in achieving true event-level segmentation. Conversely, Wang et al. [21] utilized FMCW radar echoes alongside blood oxygen saturation signals as input for an R-CNN-based respiratory event detection method. This approach directly predicted the event localization of respiratory events, demonstrating strong agreement with the AHI metric (ICC = 0.9864). Nevertheless, its reliance on blood oxygen saturation data for supplementary information prevents it from being entirely “contactless”. He et al. [22] proposed a method for blood oxygen saturation prediction based on radar-derived respiratory signals, achieving contactless SpO_2_ monitoring and elucidating the causal relationship between respiratory motion and blood oxygen saturation. Inspired by these studies, we propose a multitask-learning framework for event-level OSA segmentation. Our model simultaneously performs respiratory event prediction and blood oxygen saturation prediction tasks. This dual-task approach aims to effectively leverage the supervisory blood oxygen saturation signal to extract comprehensive respiratory information from radar echoes while also providing clinically relevant SpO_2_ predictions as a valuable reference for clinicians.

Our multitask-learning framework is designed based on the well-established physiological causal relationship between respiratory dynamics and blood oxygen saturation. During normal breathing, gas exchange between the alveoli and capillaries remains balanced, maintaining stable blood oxygen saturation levels (typically 95–100%). When apnea (complete cessation of airflow) or hypopnea (airflow reduction exceeding 30%) occurs, this balance is disrupted. Restricted airflow leads to decreased alveolar ventilation, impeded oxygen diffusion into the bloodstream, and, consequently, a decline in blood oxygen saturation. Notably, different types and degrees of abnormal respiratory events affect blood oxygen saturation levels differently: Complete apnea typically results in more significant oxygen desaturation, while hypopnea causes relatively milder decreases in blood oxygen saturation levels. Simultaneously, the event duration positively correlates with the degree of oxygen desaturation; research indicates that each additional 10 s of apnea duration can lead to an extra 1.5–2.5% decrease in blood oxygen saturation levels [23]. However, because of the oxygen storage capacity of hemoglobin and the presence of residual oxygen in the alveoli, the decline in blood oxygen saturation typically lags behind the onset of respiratory events, with a characteristic delay of 5–20 s [24]. As we employ a long window of 204.8 s, which can encompass one or multiple completely abnormal respiratory events, the model can learn more subtle respiratory feature changes from radar-captured respiratory motion data. According to this physiological mechanism, we designed a multitask-learning framework that simultaneously predicts respiratory events and blood oxygen saturation levels, using the additional supervisory signals provided by the blood oxygen saturation prediction task to guide the model in more effectively extracting respiratory-event-related features from radar signals.

The U-Net architecture has gained widespread recognition for its exceptional performance in medical image segmentation [25,26]. Its core encoder–decoder structure and skip connections provide a solid foundation for effective feature extraction and reconstruction. With further research, the application of U-Net has expanded to various domains, including time–series signal processing [12,26]. However, applying U-Net to radar signal processing and respiratory event segmentation presents unique challenges, such as adapting to the characteristics of time–series data, capturing global contextual information effectively, and leveraging multitask-learning strategies to enhance the overall model performance. To address these challenges, we propose an improved model based on U-Net, specifically designed and optimized for radar signal processing and respiratory event segmentation.

Our model retains the classical encoder–decoder structure and skip connections of U-Net while incorporating a series of key customizations to better align with the characteristics of radar signals and the requirements of respiratory event segmentation. First, to focus on time–series signal processing and reduce computational complexity, we replaced all the 2D convolutional layers in the original U-Net with 1D convolutional layers and adjusted the number of channels accordingly. Second, we upgraded the basic building blocks of the encoder–decoder by adopting a Convolution–BN-ReLU-SE Block structure. The introduction of the SE (squeeze-and-excitation) module [27] is particularly significant, as it adaptively learns the importance of each channel and dynamically adjusts channel weights. This mechanism allows the model to pay more attention to feature channels highly relevant to respiratory apnea events, thereby improving the sensitivity to abnormal-event detection. To enhance the model’s global perception, we replaced the traditional convolutional block in the bottleneck layer with a multihead self-attention module. The multihead self-attention mechanism enables feature interaction and attention enhancement across the entire context, providing the model with a global perspective, which is crucial for capturing long-range dependencies in respiratory events. To preserve temporal information, we added positional encoding before integrating the multihead self-attention module, ensuring that the model can fully utilize the temporal dimension of the input signals.

To exploit more fully the rich information embedded in radar signals and further enhance the model’s performance, we introduced a multitask-learning strategy. Specifically, we designed a blood oxygen saturation prediction branch parallel to the respiratory event prediction branch. In the model’s output layer, the blood oxygen saturation prediction branch employs a 1D convolutional layer with a kernel size of 1 and a sigmoid activation function to generate predictions for blood oxygen saturation. Through this design, we guide the encoder part of the model to encode more respiration-related features, which not only improves the event detection performance but also provides the blood oxygen saturation prediction value for the clinical diagnosis reference.

**Figure 6:**
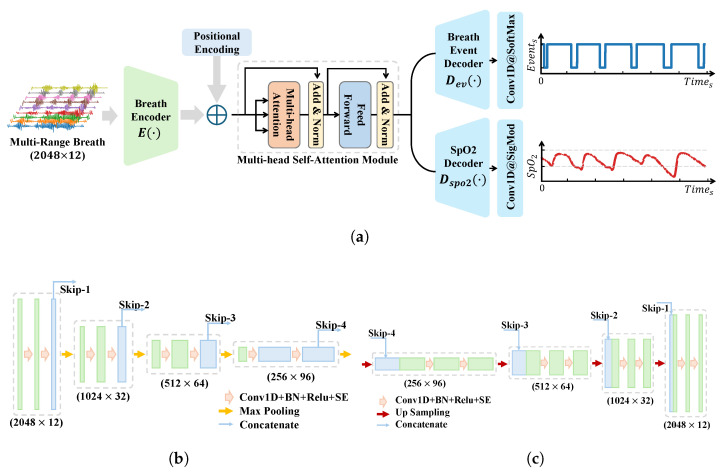
The proposed model architecture for respiratory event segmentation and blood oxygen saturation prediction. (**a**) The overall structure, including the respiratory signal encoder, multihead self-attention bottleneck, respiratory event decoder, and SpO_2_ prediction decoder, which processes multichannel respiratory signals to output respiratory event segmentation and blood oxygen saturation (SpO_2_) predictions. (**b**) The respiratory signal encoder, constructed from four convolutional blocks with downsampling and skip connections. (**c**) The decoder, also built from four convolutional blocks with upsampling and skip connections. The breath event decoder and SpO_2_ decoder are based on the same architecture but differ in their output layers.

The overall model architecture is illustrated in Figure 6a, while the detailed structures of the encoder and decoder are shown in Figure 6b,c, respectively. The model’s input is X$\in R^{N\times N_{r}}$, where *N* represents the number of sampling points, and $N_{r}$ denotes the number of respiratory channels. The model’s output consists of two parts: respiratory events, $Y_{\mathrm{event}}\in R^{N}$, and the blood oxygen saturation, $Y_{{\mathrm{SpO}}_{2}}\in R^{N}$. The respiratory event labels are equal-length sequences annotated with normal and abnormal events, while the blood oxygen saturation labels undergo a lower-bound-truncated linear scaling process, defined as follows:

(3) $$Y_{SpO_{2}}=\frac{\max(Y_{SpO_{2}}-80,0)}{20}$$

when generating the final output, the predicted blood oxygen saturation values need to be inversely mapped back to the actual blood oxygen saturation range.

The loss function design aims to balance the needs of respiratory event prediction and blood oxygen saturation prediction tasks. For the respiratory event prediction task, considering the imbalance in event labels, we selected the Dice coefficient loss function as follows:

(4) $$L_{\mathrm{Dice}}\left(\hat{y},y\right)=1-\frac{2\sum_{i=1}^{N}{\hat{y}}_{i}\;\cdot \;y_{i}+\epsilon }{\sum_{i=1}^{N}{\hat{y}}_{i}+\sum_{i=1}^{N}y_{i}+\epsilon }$$

where $\epsilon =1\times {\mathrm{10}}^{-4}$ is a smoothing factor to improve numerical stability. For the blood oxygen saturation prediction task, we designed a composite loss function as follows:

(5) $$L_{SpO_{2}}\left(\hat{y},y\right)=\frac{1}{N}\sum_{i=1}^{N}\left|{\hat{y}}_{i}-y_{i}\right|+\lambda_{\mathrm{corr}}\;\cdot \;\left(1-\frac{\sum_{i=1}^{N}\left({\hat{y}}_{i}-\bar{\hat{y}}\right)\;\cdot \;\left(y_{i}-\bar{y}\right)}{\sqrt{\sum_{i=1}^{N}{\left({\hat{y}}_{i}-\bar{\hat{y}}\right)}^{2}}\;\cdot \;\sqrt{\sum_{i=1}^{N}{\left(y_{i}-\bar{y}\right)}^{2}}}\right)$$

which combines the mean absolute error (MAE) and Pearson correlation coefficient loss, with $\lambda_{\mathrm{corr}}$ as the weighting hyperparameter. This composite loss function is designed to guide the model to learn not only the absolute values of the blood oxygen saturation but also, more importantly, the trends in blood oxygen saturation changes, thereby extracting more discriminative features and indirectly enhancing the feature encoding capability for respiratory events. The final total loss function is defined as follows:

(6) $$L_{\mathrm{Total}}\left(\hat{y},y\right)=L_{\mathrm{Dice}}+\lambda_{SpO_{2}}\;\cdot \;L_{SpO_{2}}$$

with the hyperparameter $\lambda_{SpO_{2}}$ used to balance the weights between the two task losses. We found, in the actual training, that the values of the three losses are not orders of magnitude different and are not sensitive to the change in the weight coefficients, so we set all the weights to 1.

To avoid misjudgments caused by model prediction jitter, we applied corrections to the inference results output by the model as follows: Abnormal respiratory events were merged with intervals shorter than 1 s, and abnormal respiratory events lasting less than 6 s were removed.

This chapter proposes a model based on the classical U-Net architecture, incorporating a series of modifications tailored to the characteristics of radar signals and human physiological systems. These enhancements provide unique advantages for respiratory event segmentation tasks using radar signals. First, a multitask-learning framework integrates an oxygen saturation prediction branch, offering additional supervision signals to facilitate the deeper extraction of respiratory information from radar data. Second, the effective combination of multihead self-attention mechanisms with skip connections enables the synergistic capture of global contextual information and local detailed features. Additionally, the integration of SE modules and data augmentation strategies significantly improves the model’s robustness to individual variability.

## 3. Experimental Setup

### 3.1. Dataset

To comprehensively validate the proposed method, we collected data in a polysomnography (PSG) monitoring room to construct a dataset for model training and testing. Specifically, we used the IWR6843ISK evaluation board and DCA1000 EVM data acquisition card from Texas Instruments. The data were transmitted in real time to a bedside computer and synchronized with PSG using timestamps.

Table 2 summarizes the radar parameters used in our experiments. Specifically, we configured the radar with a bandwidth of approximately 3.3 GHz, resulting in a range resolution of 4.51 cm. Reflections from the human body typically span multiple range bins. The chirp repetition frequency was set at 50 Hz, and all the data processing algorithms described in Section 2 were performed at this rate. To reduce the computational complexity while maintaining sufficient resolution for detecting respiratory events, we downsampled the data to 10 Hz before feeding them into the classification model.

We collected data from thirty-five nights, each from a different subject, including seventeen healthy individuals, thirteen with mild sleep apnea, three with moderate sleep apnea, and two with severe sleep apnea. In total, we captured 2487 complete respiratory events, with a total recording duration exceeding 320 h. The subjects were stratified by disease severity and divided into five non-overlapping subsets for cross-validation. Each recording was segmented using a window length of 204.8 s and a step size of 30 s. To address the class imbalance between normal and abnormal breathing events, we employed an adaptive sampling strategy. Specifically, when a window contained abnormal respiratory events, the step size was reduced to 15 s to increase the number of abnormal samples. This strategy is applied only in the training set, while a fixed step size of 30 s is always used in the test set. Finally, we performed fivefold cross-validation to evaluate the model’s performance in the dataset.

This approach avoided data leakage caused by overlapping windows during segmentation. Additionally, data from different individuals were strictly separated across the training and testing sets, ensuring that the reported results were based on unseen subjects.

### 3.2. Evaluation Metrics

To comprehensively evaluate the performance of the proposed algorithm with real-world data, we established two levels of metrics: event-based metrics, such as IOU, to assess the segmentation accuracy with a focus on details, and recording-based metrics, such as the respiratory event index (REI), which focuses on individual severity ratings. The combination of these two perspectives provides doctors with sufficient microscopic and macroscopic information on respiratory abnormalities for assessment.

#### 3.2.1. Event-Level Metrics

We match the ground truth events with the predicted events output by the model, considering a match to be successful if the intersection-over-union (IOU) value is greater than 0.1. Additionally, we calculate the precision, recall, and F1-score to comprehensively evaluate the model’s performance in respiratory event detection. The formulae for these metrics are as follows:

(7) $$\mathrm{Precision}=\frac{\mathrm{TP}}{\mathrm{TP}+\mathrm{FP}}\;\mathrm{Recall}=\frac{\mathrm{TP}}{\mathrm{TP}+\mathrm{FN}}$$

(8) $$F1=2\times \frac{\mathrm{Precision}\times \mathrm{Recall}}{\mathrm{Precision}+\mathrm{Recall}}\;\mathrm{IOU}=\frac{X_{\mathrm{predicted}}\cap X_{\mathrm{target}}}{X_{\mathrm{predicted}}\cup X_{\mathrm{target}}}$$

where TP represents the number of successfully matched events, FP indicates the number of falsely predicted respiratory events, and FN denotes the number of missed respiratory events. In addition, we calculate the IOU values and length prediction errors for all the respiratory events to evaluate the model’s ability to finely segment respiratory events.

#### 3.2.2. Recording-Level Metrics

For an overnight test record, the AHI is an important clinical reference metric, providing grading information on the severity of the subject’s condition. The standard method for calculating the AHI is the total number of abnormal respiratory events divided by the total sleep duration. However, in this study, we recorded the time in bed rather than the sleep time, so the AHI cannot be calculated precisely. Therefore, we used the REI as a quantitative indicator of the severity of an individual’s apnea disease. The REI is defined as the number of apnea and hypopnea events occurring per hour during the time in bed. Subsequently, we classify the severity according to the AASM standards, categorizing the record as follows: normal (0 ≤ REI < 5), mild (5 ≤ REI < 15), moderate (15 ≤ REI < 30), or severe (REI ≥ 30).

### 3.3. Comparisons

To evaluate the performance of our proposed model, we selected representative algorithms from related studies and reimplemented them for validation in our dataset. The specific representative algorithms chosen were the following:

- Base U-Net: We use the classical U-Net architecture as the baseline model and only replace 2D convolutions in the original model with 1D convolutions to fit the task data input and output;
- CNN Encoder with Multihead Attention: In [20], the authors propose a UWB-radar-based model for second-level sleep apnea detection, validated on 25 subjects. To the best of our knowledge, it achieves state-of-the-art performance among contactless radar-based sleep apnea detection methods. We reimplemented this model using the following configuration:
  – Input: Twelve-channel respiratory signals with a duration of 204.8 s;
  – Feature encoding: A CNN encoder with seven 1D convolutional layers, performing 16x downsampling;
  – Attention mechanism: Four multihead self-attention layers process the encoded features after the position-encoding layer.
  – Output: Second-level (1.6 s) classification results.
  We performed hyperparameter tuning on the number of CNN encoder layers, the number of multihead self-attention layers, and the feature-embedding dimension to achieve the optimal performance.

### 3.4. Implementation Details

All the experiments were performed on a workstation equipped with a 16-core Intel Core i7-10700K CPU, 64 GB of RAM, and an RTX 3090 GPU running the Ubuntu 20.04 operating system. All the models were implemented based on TensorFlow, version 2.8. Unless otherwise specified, the default batch size is 32, the learning rate is $10^{-5}$, and the Adam optimizer is initialized with the default parameters.

## 4. Results

### 4.1. Event-Level Performance

Table 3 presents a comprehensive comparison of event-level performances across three different methods: the classical Base U-Net [25], the CNN encoder with multihead attention (CNN–MHA) [20], and our proposed model. The evaluation metrics include the precision, recall, F1-score, and the distribution of IOU values across different ranges.

Our proposed method achieves the highest F1-score of 0.8019, demonstrating a balanced tradeoff between precision (0.8133) and recall (0.7907). This balance is crucial for clinical applications, where both false positives and false negatives must be minimized to ensure accurate and reliable diagnoses. In contrast, the CNN–MHA achieves the highest precision (0.8926) but suffers from a significantly lower recall (0.6463), resulting in a suboptimal F1-score of 0.7497. The Base U-Net performs the worst across all the metrics, with an F1-score of only 0.7279, reflecting the limitations of traditional architectures in handling long-range dependencies and complex temporal patterns inherent in respiratory signals.

The IOU distribution provides further insights into the performance of our approach. Our model shows a relatively higher concentration of IOU values in the range **0.8–1.0**, with 77.75% of the predictions falling within this interval. This suggests that our model tends to produce segmentations that align more closely with ground truth events, though there is always room for improvement. In comparison, the CNN–MHA and Base U-Net models achieve 62.46% and 60.05%, respectively, in the same IOU range, which may indicate comparatively less precise event localization.

**Figure 7:**
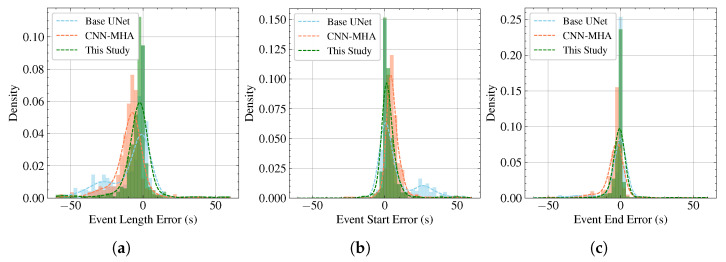
Histograms and KDE curves for different methods: (**a**) error in the duration of respiratory events; (**b**) error in the start time of respiratory events; (**c**) error in the end time of respiratory events.

Furthermore, we plotted histograms and kernel density estimation (KDE) curves to further illustrate the differences between detected events and ground truth events in terms of the start time, end time, and duration, as shown in Figure 7. The analysis reveals that the two U-Net-based methods exhibit unbiased length estimations, with error means close to zero. However, the CNN–MHA method tends to underestimate event durations. Additionally, in terms of the error concentration, the errors of the proposed method are more tightly distributed around zero compared to those of the other two methods. This indicates that the proposed method not only achieves lower detection errors but also significantly improves localization performance.

We also observe that all the methods are more accurate at predicting at the end of a respiratory event than at the beginning. This may be because OSA is often caused by upper airway collapse. As the apnea continues, the person may experience brief awakenings (micro-awakenings) because of a lack of oxygen, accompanied by body movements to adjust their sleep position. The new posture may help to open the upper airway, thus ending the apnea event and restoring ventilation. Therefore, body motion is a salient and strong feature that is easily captured using radar at the end of the apnea event. In contrast, the onset of an apnea event is a progressive upper airway collapse process that lacks this distinct, strong feature associated with body movement.

**Figure 8:**
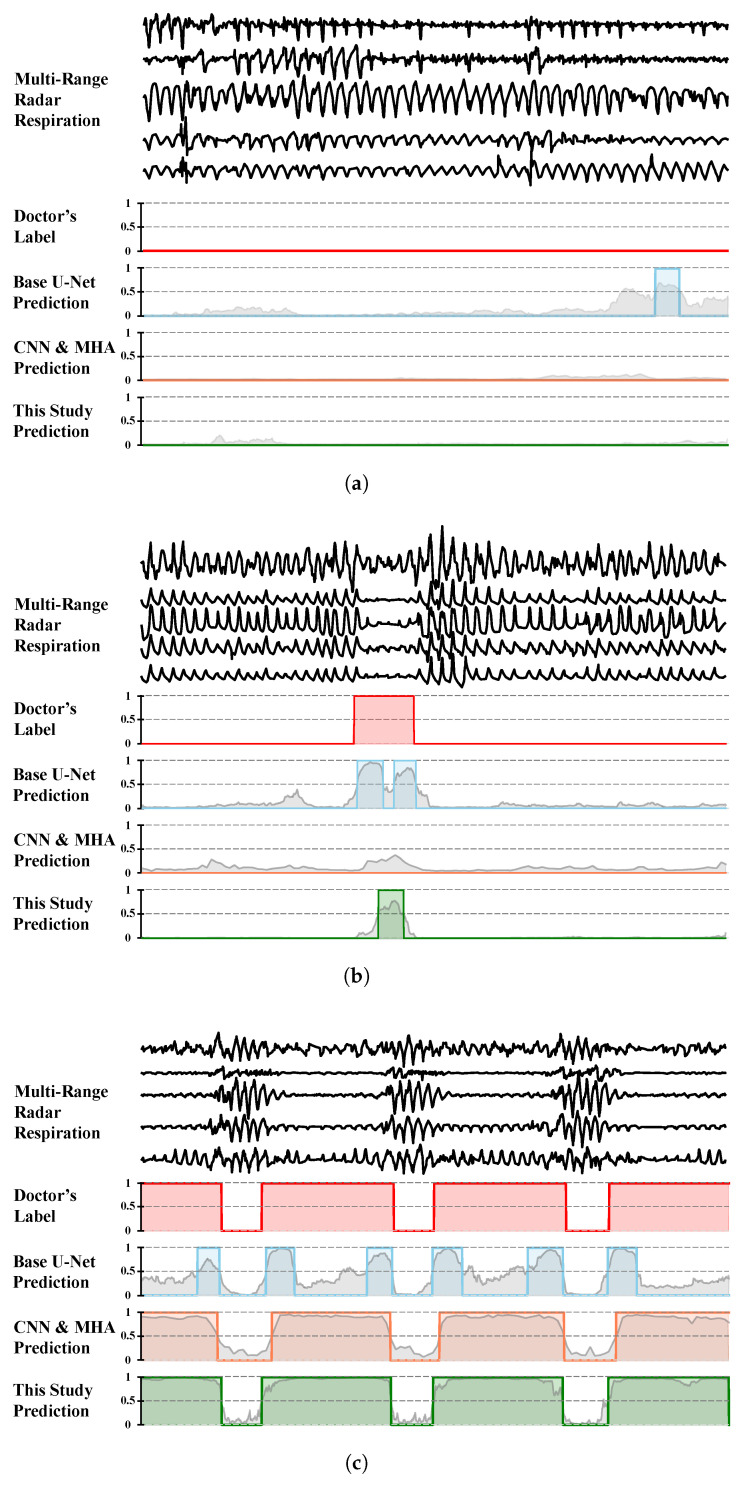
Typical signal segments from healthy people (**a**) and mild apnea (**b**) and severe apnea (**c**) patients, from top to bottom, show multichannel-radar-captured respiratory signals, doctor-labeled events based on PSG data, and probabilities/events predicted by the basic U-Net, CNN–MHA, and proposed models.

To provide an intuitive qualitative understanding of the performance differences among various methods, we present typical segments of radar-captured respiratory signals in Figure 8, along with corresponding ground truth labels and predictions from the three models.

In the normal breathing segment (Figure 8a), both the CNN–MHA method and the proposed model yield low prediction probabilities, with no false-positive events detected. In contrast, the basic U-Net model reports false positives, misclassifying normal breathing patterns as abnormal events. This observation aligns with the lower precision scores of the basic U-Net model reported in Table 3.

For patients with mild sleep apnea, occasional abnormal respiratory events occur, as illustrated in Figure 8b. As observed, both the basic U-Net model and our proposed model successfully detected these intermittent abnormal respiratory events. The basic U-Net model identified two abnormal respiratory events, while our proposed model, although accurate in detecting the correct number of events, tended to underestimate their duration. The CNN–MHA model demonstrated the poorest performance in this case study, exhibiting missed detections. This limitation renders it as unsuitable for screening applications, where false negatives present a significant clinical risk by failing to identify affected individuals who require intervention.

For the segment with recurring apnea events (Figure 8b), all three methods successfully detected the presence of abnormal breathing events, which is critical for OSA screening. However, compared to the basic U-Net, the proposed method and the CNN–MHA method demonstrate superior performances in delineating event boundaries. These two methods more closely match the doctors’ annotations in terms of event start and end times, indicating more accurate localization of respiratory events. Although the CNN–MHA method also captures events accurately, its predicted event boundaries appear less refined compared to those of the proposed method. Additionally, although the basic U-Net detects events, its less distinct probability outputs lead to inaccuracies in event counting and boundary detection.

### 4.2. Recording-Level Performance

In addition to the precise segmentation and localization of respiratory events, evaluating the overall performance of complete recordings is equally crucial. This directly impacts whether a subject is diagnosed with sleep apnea and the severity grading. In Table 4, we compare the performances of the three methods in apnea severity grading and REI prediction. The results show that all three methods achieve an accuracy rate above 80%, while our proposed method achieves accuracy, precision, recall, and F1-scores exceeding 90%, demonstrating its high performance in severity grading. Furthermore, the proposed method achieves the lowest mean absolute error (MAE) of 1.76 events/hour in REI prediction. Because most subjects have an REI within the range 0–20 events/hour, accurately handling the boundaries of low REI intervals is the key for achieving high performance, whereas identifying patients with an REI exceeding 30 events/hour is relatively easier and more accurate.

We generated confusion matrices, scatter plots, and Bland–Altman analysis graphs for the predicted REI values of each method. The results indicate that all three methods exhibited high degrees of correlation but differed significantly in the intercepts of their fitted lines. From the Bland–Altman plots, it can be observed that the proposed method has fewer outliers, while the baseline U-Net model and CNN–MHA model not only produce more outliers but also exhibit larger mean errors. We further analyzed the ability of the model to classify samples with different severity levels by confusion matrices. It can be seen that all the models have a poor ability to classify healthy and mild sleep apnea patients, while—except for one moderate sleep apnea patient misclassified by CNN–MHA as severe—the other two models successfully classified all the moderate and severe sleep apnea patients. Overall, all three methods achieved acceptable performances in the severity grading task, with the primary differences lying in their abilities to segment respiratory event details.

**Figure 9:**
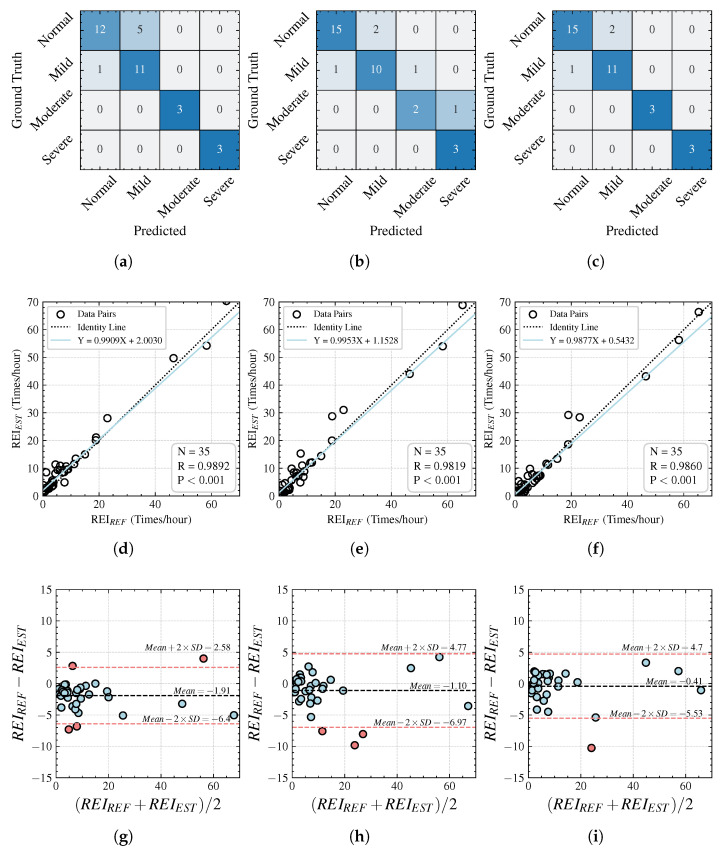
Confusion matrices, scatter plots, and Bland–Altman analysis graphs for recording-level analyses: (**a**,**d**,**g**) for basic U-Net; (**b**,**e**,**h**) for CNN–MHA; (**c**,**f**,**i**) for this study.

We further consider potential challenges in clinical applications. One aspect involves the monitoring accuracy across different ages, BMIs, and sleep postures. The fundamental principle of millimeter-wave radar respiratory monitoring is the measurement of skin surface displacement. Respiratory movements cause thoracic and abdominal cavity contraction and expansion, manifesting as displacement changes detected by radar. Because of variations in BMI, age, or sleep position, respiratory intensity differs among subjects. Those with stronger respiration typically generate larger signal amplitudes, with more sinusoidal respiratory signals detectable across multiple distance units; meanwhile, subjects with weaker respiration produce steadier signals that may only present stable waveforms in limited distance units, appearing as noise in others. During data acquisition, we did not apply any exclusion criteria, nor did we restrict subjects’ sleep positions; thus, our dataset encompasses diverse data that are representative of the actual population’s characteristics. Because we utilized multichannel respiratory waveforms as model inputs and trained the model with diverse data, the model does not exhibit significant performance disparities across individuals. This is shown in the Bland–Altman agreement analysis in Figure 9, where the distribution of REI prediction errors across different individuals remains consistent. Furthermore, related preliminary research [28] indicates that radar-based nocturnal vital sign monitoring is insensitive to variations in BMI, age, and sleep posture, without significant performance differences.

Another consideration is whether the proposed model’s performance satisfies the requirements for two primary scenarios: sleep apnea screening and treatment efficacy monitoring. For initial screening tools, our model’s performance is sufficiently acceptable. For instance, a clinical study [29] demonstrated that portable-respiratory-monitoring devices typically achieve sensitivities between 85 and 95% for grading of the severity, while our model reached 91.43%. In practice, certain false positives are acceptable, as positive results are generally confirmed by PSG. In home monitoring scenarios, high false-positive rates may induce unnecessary anxiety; however, for tracking long-term treatment efficacy trends, the impact of occasional false positives would be averaged over time. Considering its non-contact measurement advantages in comfort, our approach is more likely to be selected as a long-term at-home monitoring tool for tracking treatment outcomes. Therefore, the proposed model satisfies the requirements for clinical screening and treatment efficacy monitoring, with its high accuracy rate and non-contact advantages facilitating its clinical implementation.

## 5. Discussion

### 5.1. Ablation Studies

The SE module and the multihead self-attention (MHSA) layer at the bottleneck are the key components of the proposed model (with the auxiliary task branch discussed in the next section). To validate the effectiveness of these components, we designed a series of ablation experiments. The performances of the different models are summarized in Table 5, where we examine the impact of each individual component as well as their combined effect.

From the results in Table 5, it is evident that the attention mechanism plays a crucial role in the model’s performance. Specifically, the inclusion of either the SE module or the multihead self-attention layer leads to a noticeable improvement in the performance. When both components are utilized together, the model achieves the best performance overall. This can be attributed to the complementary functions of these two modules. The SE module helps the model to identify which respiratory channels are more important for event detection, allowing it to focus on the most relevant features. On the other hand, the multihead self-attention layer provides the model with a global view, enabling it to better capture the contextual information of the respiratory signals and focus on abnormal breathing segments that are critical for detecting events.

Overall, the ablation studies demonstrate that both the SE module and the multihead self-attention layer are indispensable for achieving the high performance observed in our proposed model. These components work synergistically to improve the model’s ability to detect and localize respiratory events, which is crucial for applications such as sleep apnea monitoring.

**Figure 10:**
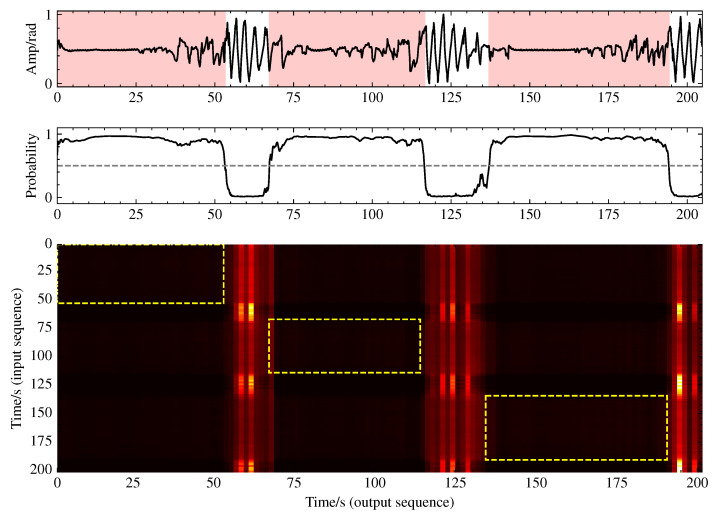
Visualization of multihead self-attention weights revealing normal breathing reference patterns.

To gain deeper insight into the functionality of the multihead self-attention mechanism, we conducted a visualization analysis of its attention weights. As illustrated in Figure 10, the attention distribution exhibits pronounced vertical stripe patterns rather than the typical diagonal patterns, with these vertical stripes precisely aligning with normal breathing segments. This indicates that the model establishes a ’reference framework’ based on normal breathing, comparing respiratory patterns from all the time points against these normal templates to identify anomalous events. This comparative analytical approach simulates the diagnostic process employed by human experts, elucidating the significance of the multihead self-attention component in the bottleneck layer for sleep apnea event detection.

### 5.2. Analysis of $SpO_{2}$ Prediction Task

We evaluate the effectiveness of the SpO_2_ prediction auxiliary task from two perspectives: first, the impact of introducing the SpO_2_ prediction task on the model’s ability to recognize respiratory events; second, the influence of the design methodology of the SpO_2_ prediction auxiliary task itself, such as the composite loss function’s design. To this end, we conducted a series of experiments, with the results shown in Table 6.

**Figure 11:**
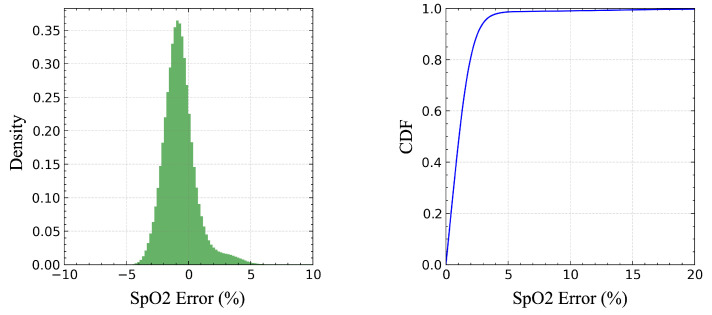
Error distribution and cumulative error distribution for SpO_2_ prediction.

The table reveals that introducing the SpO_2_ auxiliary task leads to an improvement in the F1-score of 0.99%, indicating that adding SpO_2_ supervision signals helps the model to extract more features related to respiratory events. The SpO_2_ branch achieves an MAE of 1.41% for SpO_2_ prediction, with a Pearson correlation coefficient of 0.45. The error distribution and cumulative error distribution are shown in Figure 11. Its performance is comparable to that of reflective SpO_2_ measurement methods, such as Apple Watch’s SpO_2_ measurements, which have an MAE of around 2.8% [30], thereby reaching a usable level.

**Figure 12:**
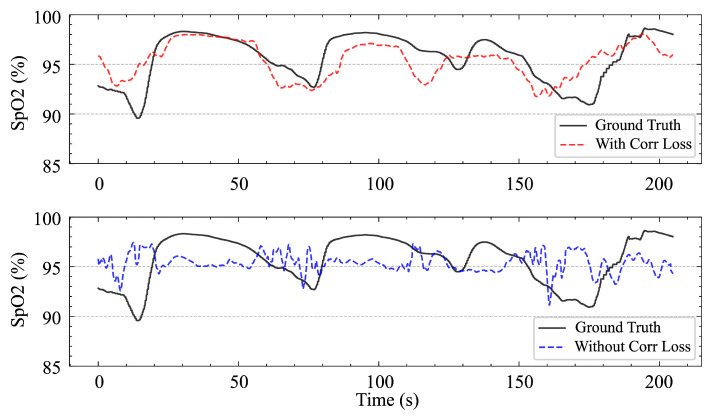
SpO_2_ predictions with and without the use of correlated losses.

However, the success of the SpO_2_ branch also relies on the use of composite loss functions. Without incorporating relevant loss terms, the model tends to predict the mean value, which—although it results in a lower MAE—provides no benefit to the event classification task and produces SpO_2_ waveforms with a very low correlation degree, rendering them of little reference value, as shown in Figure 12. This can be explained by the fact that the fluctuation in SpO_2_ is the signal that is the most directly related to respiratory events, and there is no relationship with the absolute magnitude of SpO_2_.

Using the composite loss function also has another effect. As shown in Figure 11, the error distribution of the blood oxygen saturation prediction is slightly skewed toward negative values; that is, the model tends to slightly underestimate the blood oxygen saturation value, while the model shows a symmetric error distribution when using only the MAE loss. Considering the performance gain from the introduction of the correlation loss, we consider this deviation to be acceptable. In addition, from a clinical perspective, underestimating blood oxygen saturation is actually more conservative and safer than overestimating blood oxygen saturation. The overestimation of the blood oxygen saturation may lead to the ignorance of potential hypoxic risks, whereas underestimation increases vigilance and prompts clinicians to pay more attention to possible problems.

We further analyzed the tradeoffs between the computational costs and benefits associated with introducing the SpO_2_ auxiliary task. First, adding the SpO_2_ prediction branch indeed increased the computational burden, albeit to a limited extent. Specifically, the inference time increased from 8.1 ms/sample to 9.5 ms/sample, representing an approximately 17.3% increase. The training time increased by approximately 20%, though this constitutes a one-time cost with minimal impact on deployment. Regarding the benefits, incorporating the SpO_2_ auxiliary task resulted in an improvement in the F1-score of approximately 1.0% (from 0.7920 to 0.8019), while the proportion of samples with IOU values of >0.8 increased from 75.45% to 77.75%, indicating enhanced event boundary localization precision. Additionally, SpO_2_ prediction serves as a “free” supplementary output with significant clinical reference value. In sleep apnea diagnoses, oxygen saturation represents a critical indicator for assessing disease severity. Considering these factors comprehensively, we conclude that the performance improvements and clinical utility provided by the SpO_2_ auxiliary task substantially outweigh its moderate computational cost increase, particularly in sleep-monitoring applications where real-time processing is not essential.

### 5.3. Annotation Consistency Analysis

**Figure 13:**
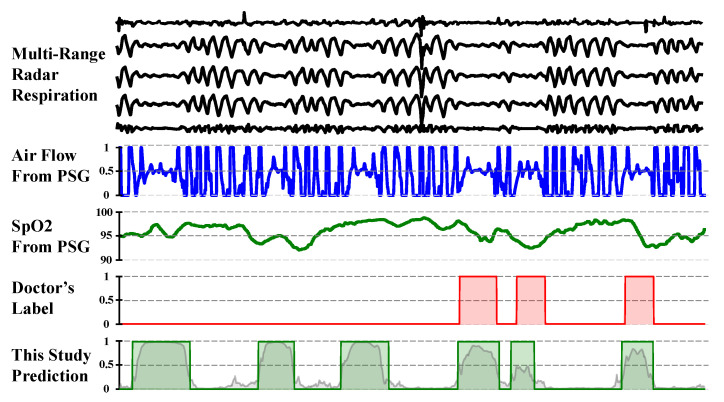
Comparison of model and physician annotations. From top to bottom: radar multichannel breathing waves; PSG nasal airflow and SpO_2_; doctor’s labels and model predictions.

During the result analysis, we observed a noteworthy phenomenon: although the model was trained using physician annotations as the gold standard, in some cases, its predictions were closer to the underlying true values than the physician annotations, as shown in Figure 13. We noted that during periods of frequent apnea events, physicians tended to conservatively annotate normal breathing, potentially over-labeling abnormal respiratory segments. Upon analysis, we found that physician annotations were not entirely accurate, possibly because of variations in guideline interpretation, differences in experience, and fatigue from prolonged work, leading to annotation errors and inconsistencies. Despite the presence of “noise” in the supervision signal used for model training, the model appeared to learn robust patterns from large-scale data, mitigating the impact of the label noise and producing estimates closer to the underlying physiological truth. Similar phenomena have been reported in studies on automatic X-ray image annotation [31] and automated sleep staging [32].

This observation suggests that automated model-based annotation can effectively reduce subjective human interference, improving the consistency and potential accuracy in annotations, thereby significantly enhancing medical efficiency. In the fields of sleep apnea screening and treatment evaluation, the application of automated annotation technology holds promise for providing more consistent and precise results, facilitating large-scale screening efforts.

### 5.4. Limitations

Although this study evaluated the model’s performance using non-repeated individual tests, its generalizability to novel environments requires further validation because of limitations in the data collection conditions and dataset size. Therefore, expanding the dataset and enhancing its diversity with respect to environmental and individual variability constitutes a critical direction for future research. Furthermore, the current investigation focused exclusively on the localization of abnormal respiratory events without differentiating between specific subtypes, such as obstructive apnea, central apnea, and hypopnea. Finally, this study used the REI rather than the AHI as a quantitative indicator of sleep apnea severity, which may underestimate the severity of sleep-disordered breathing, as the time in bed is typically longer than the actual sleep time. However, this methodological limitation also exists in most at-home portable-sleep-monitoring devices.

Multimodal fusion technology represents a promising approach to address this limitation. For instance, acoustic signals could be utilized to monitor snoring patterns, while video signals might capture more granular chest and abdominal movement dynamics. These non-contact measurement means are suitable for radar combination to enhance the sensing capability. However, the incorporation of such additional modalities potentially introduces privacy concerns that must be carefully evaluated within specific application contexts. Future work will focus on combining multimodal fusion techniques with extended datasets to advance the subtyping of abnormal respiratory events during sleep and the more precise estimation of sleep durations.

## 6. Conclusions

This study introduces an event-level sleep apnea detection method using FMCW radar, effectively solving the research problem of achieving precise, event-level OSA diagnoses using non-contact technology. Our main contributions include the following: (1) to the best of our knowledge, the first reported event-level segmentation of sleep apnea from FMCW radar signals, providing detailed diagnostic information; (2) an optimized U-Net-based deep-learning model incorporating multihead self-attention and SE modules, tailored for radar signal processing; and (3) a multitask-learning framework with auxiliary SpO_2_ prediction, enhancing the model’s performance and clinical relevance.

Validation in a real-world clinical dataset containing the data of 35 subjects demonstrated the superior performance of our approach, achieving an F1-score of 0.8019 for event-level segmentation and 91.43% accuracy in OSA severity grading. These results underscore the significant potential of our radar-based system for contactless, low-cost, and accurate sleep apnea assessment. This technology paves the way for large-scale, non-contact OSA screening and long-term treatment efficacy monitoring, offering a convenient and comfortable solution. Future work will focus on expanding the dataset diversity and exploring event subtyping, further realizing the clinical translation of radar-based sleep health monitoring. This research provides a crucial step toward accessible and effective OSA management, contributing to improved public health outcomes.

## Figures and Tables

**Table 2:** Radar parameters.

Parameter	Value	Parameter	Value
Start Frequency	60 GHz	Range Resolution	4.51 cm
Chirp Slope	65 MHz/μs	Chirp Rate	50 chirps/second
ADC Sample Rate	5 MHz	Transmission Power	10 dBm
Number of ADC Samples	256	Bandwidth	3.3 GHz

**Table 3:** Performance comparison of different methods in respiratory event segmentation.

Model	Precision	Recall	F1	IOU			
				0.0–0.3	0.3–0.6	0.6–0.8	0.8–1.0
**Base U-Net [25]**	0.7022	0.7556	0.7279	0.0539	0.1887	0.1569	0.6005
**CNN–MHA [20]**	**0.8926**	0.6463	0.7497	0.0229	0.0917	0.2607	0.6246
**This Study**	0.8133	**0.7907**	**0.8019**	0.0164	0.0656	0.1405	0.7775

**Table 4:** Performance comparison of methods for REI estimation and severity grading.

Method	Accuracy	Sensitivity	Precision	F1-Score	REI MAE
**Base U-Net**	83%	83%	86%	0.83	2.2955
**CNN–MHA**	86%	86%	86%	0.86	2.0858
**This Study**	**91%**	**91%**	**92%**	**0.91**	**1.7630**

**Table 5:** Comparison of the ablation experimental results.

Model	Precision	Recall	F1	IOU			
				0.0–0.3	0.3–0.6	0.6–0.8	0.8–1.0
**SE+MHSA**	0.8133	0.7907	0.8019	0.0164	0.0656	0.1405	0.7775
**SE**	0.8024	0.7444	0.7723	0.0199	0.1119	0.2637	0.6045
**MHSA**	0.7896	0.7852	0.7874	0.0142	0.0590	0.1533	0.7736
**None**	0.6903	0.6315	0.6596	0.0587	0.1965	0.2933	0.4516

**Table 6:** Results of ablation experiments for the SpO_2_ prediction auxiliary task: M1—model without SpO_2_ prediction task; M2—model without corr loss.

Model	Precision	Recall	F1	IOU				SpO_2_ Prediction	
				0.0–0.3	0.3–0.6	0.6–0.8	0.8–1.0	MAE	Corr
**Full**	0.8133	0.7907	0.8019	0.0164	0.0656	0.1405	0.7775	1.41%	0.45
**M1**	0.8102	0.7746	0.7920	0.0527	0.0592	0.1336	0.7545	-	-
**M2**	0.8082	0.7802	0.7940	0.0114	0.1090	0.1233	0.7563	1.52%	0.12

## Data Availability

The data presented in this study are available on request from the corresponding author due to the protection of participants’ privacy.
